# Higher Diet Quality Does Not Predict Lower Medicare Costs but Does Predict Number of Claims in Mid-Aged Australian Women

**DOI:** 10.3390/nu3010040

**Published:** 2011-01-07

**Authors:** Clare E. Collins, Amanda Patterson, David Fitzgerald

**Affiliations:** 1 Nutrition and Dietetics, School of Health Sciences, University of Newcastle, HA12, Hunter Building, University Drive, Callaghan, NSW 2308, Australia; Email: Amanda.Patterson@newcastle.edu.au; 2 Australian Longitudinal Study on Women’s Health, School of Population Health, University of Queensland, Herston Road, Herston QLD 4006, Australia; Email: d.fitzgerald@sph.uq.edu.au

**Keywords:** diet quality, diet scores, diet index, nutrition surveys, Medicare, health costs

## Abstract

Optimal dietary quality, indicated by higher diet quality index scores, reflects greater adherence to National dietary recommendations and is also associated with lower morbidity and mortality from chronic disease. Whether this is reflected in lower health care cost over time has rarely been examined. The aim of this study was to examine whether higher diet quality, as measured by the Australian Recommended Food Score (ARFS), was associated with lower health care costs within the mid-aged cohort of the Australian Longitudinal Study on Women’s Health. We found that there was a statistically significant association between five year cumulative costs and ARFS, but in the opposite direction to that predicted, with those in the highest quintiles of ARFS having higher health care costs. However the number of Medicare claims over the six year period (2002–2007) was lower for those in the highest compared with the lowest quintile, *p* = 0.002. There is a need to monitor both costs and claims over time to examine health care usage in the longer term in order to determine whether savings are eventually obtained for those with the dietary patterns that adhere more closely to National recommendations.

## 1. Introduction

Numerous indices of dietary quality highlight that higher scores, reflecting greater adherence to National Dietary Recommendations, are commonly associated with important reductions in morbidity and mortality [1]. However, this has not been completely consistent across the range of study populations, the range of tools developed to capture diet quality, variety and/or adherence to National dietary guidelines, or the range of outcome indicators of morbidity and mortality [1]. In addition, the association in some studies has been less strong for women [1]. 

Kant [2] examined the association between nutrient intakes with age and self-reported chronic medical conditions, defined as those that a doctor had advised them they had, in a nationally representative sample of subjects (*n* = 7207) aged greater than 25 years in the Continuing Survey of Food Intakes by Individuals, 1989–1991. Age and self-reported medical conditions were associated with an increased risk of not consuming 100% of the Recommended Dietary Allowance (RDA) for some nutrients (vitamins E, B12 and calcium, zinc and iron, *p* < 0.05). Women were more likely to report nutrient intakes that were less than 100% RDA compared with men, but for women this was not related to age or morbidity as it was for the men.

Mortality is an objective and final outcome by which a tool measuring diet quality and variety can be validated. However, intermediate associations with early indices of morbidity, including health service use or health care costs, may be important and offer opportunities to develop and test health promotion interventions aimed at improving diet quality and reducing morbidity and mortality. Additionally, they may serve as motivators for individuals to improve their dietary intake and reduce morbidity. 

Internationally there have only been limited studies that have prospectively examined the relationship between diet quality scores and health care costs. Daviglus demonstrated that in 1063 men from the Chicago Western Electric Study, those in the highest tertile of fruit and fruit plus vegetable intakes during middle age had lower mean annual and cumulative Medicare charges over a 15 year period [3]. They found that after adjusting for baseline age, education, total energy intake and baseline risk factors, high fruit and vegetable intakes were associated with lower mean annual and cumulative Medicare charges (including total charges) and CVD- and cancer-related charges, although the *P* value for trend ranged from 0.019 to 0.862. While many of the results were not statistically significant, the trends do suggest that having higher fruit and vegetable intakes earlier in adulthood has the potential for lower health care costs in older age as well as improved health status.

Other than this analysis by Daviglus, we were not able to locate any other studies that have examined associations between diet quality and subsequent cost of health service use. In the USA Medicare is a social insurance program administered by the US government providing health insurance cover to people who are aged 65 and over or who meet other special criteria. It covers hospital-related inpatient and outpatient services and skilled nursing facility services. Outpatient charges encompass emergency room visits, clinic and ambulatory surgery, laboratory tests, radiography, rehabilitation therapy, radiation therapy and renal dialysis. In Australia, Medicare Australia (MA) administers a universal health care program called Medicare. It is funded by income tax and an income-related Medicare levy. Medicare is the largest source of primary health care spending in Australia, and covers the scheduled fees for out-of-hospital services for doctors, including specialist consultation fees and tests and examinations ordered by doctors, (e.g., X-rays and pathology tests) as well as eye tests performed by optometrists. It also covers most surgical and other therapeutic procedures performed by doctors, some surgical procedures performed by approved dentists, specified items under the Cleft Lip and Palate Scheme, and specified items for allied health services as part of the Enhanced Primary Care (EPC) program. In-hospital services covered as a public patient include treatment by doctors and specialists nominated by the hospital. MA also administers a separate Pharmaceutical Benefits Scheme (PBS), which makes a range of prescription medicines available at affordable prices to Australian residents. The aim of this study is to examine associations between diet quality as measured by the Australian Recommended Food Score (ARFS) and indicators of health status, health service use and Medicare costs within the mid-aged cohort of the ALSWH.

## 2. Methods

The data for this research comes from the Australian Longitudinal Study on Women’s Health (ALSWH) which was established to investigate multiple factors affecting the health and wellbeing of women over a 20-year period. Women in three age groups (“younger” 18–23, “mid-aged” 45–50 and “older” 70–75 years) were randomly selected from the national health insurance database (Medicare) which includes all permanent residents of Australia. There was over-representation of women living in rural and remote areas to allow sufficient numbers of these women for reliable data. The methods for ALSWH have been previously published in detail [4,5,6]. 

## 3. Assessment of Dietary Intake

Dietary intake was assessed using the Dietary Questionnaire for Epidemiological Studies (DQES). The DQES asks respondents to report their usual consumption of 74 foods and six alcoholic beverages over the preceding 12 months using a 10-point frequency option from ‘never’ up to ‘three or four times per day. Portion size photographs are used to adjust the serve size for vegetables, meat and casseroles. Additional questions are asked about total number of daily serves of fruit, vegetables, bread, dairy products, eggs, fat spreads and sugar, as well asking the type of bread, dairy products and fat spreads used. Nutrient intakes are computed from NUTTAB 1995, a food composition database of Australian foods [7], using software developed by the Cancer Council of Victoria. Both the development of the DQES [8] and its validation in mid-aged Australian women have been previously reported [9].

## 4. Subjects

Data used in this study has been derived from the mid-aged cohort of the Australian Longitudinal Study of Women’s Health (ALSWH). Survey 1 (*n* = 13,716) was conducted in 1996 and the respondents have been shown to be broadly representative of the national population of women in the target age groups [5]. Survey 2 (*n* = 12,338) was conducted in 1998 and Survey 3 (*n* = 11,228) was conducted in 2001. Data from the FFQ included in Survey 3 are the focus of this paper.

The response rate for Survey 3 of the mid-aged cohort was 83% of women who had completed Survey 1 and had not died or become too ill to complete further surveys. The non-respondents included those who did not complete Survey 3 (7.4%), withdrew from the study completely (2.8%) or could not be contacted (6.8%) [6]. From the women who completed Survey 3 (then aged 50–55 years), 11,194 completed a usable FFQ. 

## 5. Australian Recommended Food Score (ARFS)

The ARFS was modelled on the Recommended Food Score by Kant and Thompson [10] and was calculated based on DQES items consistent with national recommendations in the Dietary Guidelines for Australian Adults [11] and the core foods given in the Australian Guide to Health Eating (AGHE) [12]. The ARFS methodology has been reported in detail elsewhere [13]. Briefly, items consumed less than once a week scored zero and those consumed once a week or more scored one. A point was added for each of the questions on type and amount of core foods based on the following; at least two fruit serves per day, at least four vegetable serves per day, using reduced fat or skimmed milk, using soy milk, consuming at least 500 mL of milk per day, using high fibre, wholemeal, rye or multigrain breads, having at least four slices of bread per day, using polyunsaturated or monounsaturated spreads or no fat spread, having one or two eggs per week, using ricotta or cottage cheese, using low fat cheese. A maximum of two points was added for alcohol consumption: one point for moderate frequency and the second point for moderate quantity, when they drank alcohol. FFQs that had greater than four missing values were discarded and for those with less missing values were recoded to zero. The maximum ARFS possible is 74. The ARFS was divided into quintiles to create a categorical variable with quintile one representing the lowest dietary quality score and quintile five the highest or best score. 

## 6. Medicare Data

The health services utilization and cost data is provided by Medicare Australia. In the third survey of the mid-aged cohort, 7091 out of the 11,226 women (63%) gave consent for linkage of their Medicare data to their survey. There were significant but small differences between consenters and non-consenters by area of residence, and those consenting to Medicare linkage tended to be better educated, more likely to be able to manage on their available income and more likely to say their health was excellent, very good or good [14].

Only ALSWH respondents who consented to link their Medicare data were used in this analysis. There were 6410 women who had given both Medicare consent and had an ARFS. All Medicare data were collected for the five years from 2002 to 2006, with data for only the number of claims also available for 2007. Annual data was totalled for each woman to determine the amount the women spent on Medicare health care and the number of health care claims. Women with no claims for a particular year do not appear in our data as Medicare Australia only provides data for women for which there are actual charges. The “charge” item was used as the main outcome variable. This is the total cost of the treatment. There are two other Medicare variables, the Benefit and the Gap. The Benefit is what was paid back to the patient, while the Gap is the difference between the Charge and the Benefit (*i.e.*, what the patient paid out of their own pocket). The Charge is almost always higher than the Benefit. The upper and lower 1% of charges was deleted to avoid extreme values. The distribution of the charges is highly skewed to the right. Therefore, the remaining data were transformed by taking the natural log of the Charges, Benefits and Gap. The Medicare data used in the study were from the Medicare Benefits Schedule of MA and not from the Pharmaceutical Benefits Scheme. 

## 7. Statistics

Data manipulation and statistical analyses were performed used SAS [15]. Generalised Linear Modelling was performed in SAS using Proc GLM with area of residence and educational attainment used as covariates in all statistical modelling to adjust for the sampling frame and for socioeconomic status. *P*-values < 0.05 were considered statistically significant. Tests of association were conducted using Chi-squared analysis.

## 8. Results

The ARFS was normally distributed while the Medicare costs and claims were not. At Survey 3 there was no association between quintiles of ARFS and the 2001 Medicare Charge, after controlling for education level and area of residence (*p* > 0.05). 

The median five year (2002–2006) cumulative Medicare Charge, Benefit and Gap costs and the six year (2002–2007) cumulative number of Medicare claims were examined by quintiles of ARFS with a mean (sd) ARFS of 21.0 (4.4) for quintile 1 and 45.8 (3.4) for quintile 5. Table 1 indicates that there was no significant trend between Benefits by quintile, but there was a statistically significant association between Charges and Gap, and ARFS. Compared with quintile 1, all cumulative Charges, Benefits and Gap are higher in quintile 5, with the highest Charge and Benefit related to quintile 3. 

**Table 1:** Median five year (2002–2006) cumulative Medicare costs ($AUS) for mid-aged Australian women by quintile of ARFS, where 1 = lowest and 5 = highest quintile.

ARFS quintile	ARFS mean (SD)	Charge $	95% Confidence Limits		Benefit $	95% Confidence Limits		Gap $	95% Confidence Limits	
		*N* = 6223			*N* = 6223			*N* = 6094		
1	21.0 (4.4)	2867	2736	2867	2254	2156	2356	460	428	495
2	29.1 (1.4)	2854	2713	2854	2182	2079	2290	532	492	575
3	34.1 (1.4)	3132	2989	3132	2384	2280	2492	589	548	633
4	38.9 (1.4)	2907	2761	2907	2194	2089	2304	588	543	636
5	45.8 (3.4)	3077	2923	3077	2311	2200	2427	619	571	670

*P* value for ARFS quintile; Charge *P* = 0.018; Benefit *P* = 0.047; Gap *P*< 0.0001.

The modelling was extended to include the total number of Medicare claims (not dollar costs) from 2002 to 2007 inclusive and the ARFS quintiles were significantly related to the number of claims (*p* = 0.002). The lowest ARFS quintile’s mean (95% CI) number of claims was 110 (105–115) and the highest ARFS quintile’s number of claims was 100 (95–106). A difference of means test shows this is significantly different (*p* = 0.0011). 

## 9. ARFS Subscales

To examine whether the ARFS sub-scales performed differentially to the total score, they were examined individually with cumulative Medicare Charges. Table 2 reports the adjusted *P* values for the models in which ARFS subscale score was used and the outcome variable was the log-transformed five year cumulative Medicare Charge. 

**Table 2:** Generalised Linear Models of five year cumulative Medicare Charges for mid‑aged Australian women, adjusted for area of residence and educational attainment, against sub-scale component scores of the Australian Recommended Food Score (ARFS), where 1 = lowest and 5 = highest quintile.

ARFS subscale	Mean $ (95% CI) Lowest subscale score	Mean $ (95% CI) Highest subscale score	*P*-values for ARFS
Fruit	2700 (2420, 3012)	3076 (2676, 3536)	0.09
Vegetable	5025 (3575, 7062)	2814 (2287, 3462)	0.07
Dairy	2592 (2261, 2971)	3326 (2501, 4422)	<0.0001
Grain	2972 (2516, 3510)	2972 (2516, 3510)	0.58
Fat	2798 (2558, 3060)	3471 (2973, 4052)	<0.0001
Meat	2959 (2753, 3180)	2897 (2558, 3281)	0.42
Fish	2851 (2751, 2954)	3003 (2872, 3140)	0.015
Nuts and Beans	3011 (2902, 3124)	3207 (1852, 5553)	0.90
Alcohol	3081 (2923, 3247)	2949 (2864, 3037)	0.29

*N* = 6223 for all subscales, except alcohol where *N* = 6212.

## 10. Discussion

This is the first study of cumulative Medicare costs and claims in Australia in relation to diet quality in mid-aged Australian women. While we have previously reported associations between diet quality as measured by the ARFS and self-reported health status and indices of health service usage [13], this was not mirrored in association with the Medicare Charge or reflected in the cumulative Medicare Charge prospectively over 5 years, but was associated with a reduced number of claims. These data suggest that Medicare costs, including the Medicare Charge, Benefit and Gap, increase rather than decrease from the lowest compared with the higher quintiles of diet quality and that each claim must be more costly. The relationship with cost is in the opposite direction to our hypothesis, although there was a 5-year cumulative difference of only $AUS 110 from the bottom to the top quintile, and the trend was not consistent across quintiles. This relationship is likely to be confounded by charges incurred for routine screening services (e.g., cervical and breast cancer screening) as women with higher ARFS scores are possibly more diligent with regards to screening. Even though AFRS was adjusted for educational attainment there may be residual confounding, particularly given the relationship previously demonstrated with higher education [13]. Five years may not be a long enough time period for any potential Medicare savings to be evident. An additional important consideration in relation to the higher Gap incurred by women who have a higher ARFS is that they may access health services that have a higher cost, by their own choice.

Interestingly our data did demonstrate highly significant relationships with both the dairy and fat subscales, but once again in the opposite direction to that predicted. That Medicare charges were increased with higher ARFS sub-scale scores for the dairy, fish and healthy fats groups, which are markers of regular consumption of a greater variety of healthy choices, is a concern. This suggests that adhering to National dietary recommendations to optimize health [11] is associated with greater health care costs. Clearly longer follow-up will be important to ascertain these costs over a longer timeframe. When the ARFS fruit and vegetable sub-scales were examined there was no significant relationship but there was a trend towards higher Medicare charges in association with a low vegetable intake. This finding compares to the results shown by Daviglus [3] who followed up male employees from the Western Electric company in the USA after 25 years and demonstrated lower Medicare costs for those with the highest reported fruit and vegetables intakes. Although there are differences in the way the scales are coded and that this male population is also older (>65 years), collectively it does suggest that promoting more frequent consumption of a greater variety of vegetables may be a future important public health strategy if this trend becomes significant after longer follow-up.

The differences with the current study could be further explained by the relatively short five year follow-up period for this group and that our study is in women. This does suggest it may be worthwhile to continue to cumulate further Medicare data over a much longer time period to examine whether there will eventually be a Medicare cost saving and at what time point it may become apparent. Particularly as a recent review has highlighted that higher dietary quality is consistently inversely related to all cause mortality, with a protective effect of moderate magnitude [1], although associations appear to be stronger for men, especially for all cause and CVD mortality. 

There are a number of important limitations in this analysis. Baseline health status was not considered, although the top and bottom one percent of extreme values were removed to adjust for this. Not all costs related to medical treatments will be captured by the Medicare Australia data, although one could hypothesize that those of higher socio-economic status may be more likely to seek additional medical treatment that is not covered by Medicare. In addition, the dollar costs of Medicare are relatively unstable and influenced by a small number of large expenses. Further, a comparison of consenters and non-consenters [14] indicated that there were significant but small differences according to area of residence and consenters were more likely to say their health was excellent, very good or good than non-consenters, which limits the generalizability of the findings. Although misclassification with ARFS quintiles may affect the ability to detect trends related to Medicare costs, the ARFS was modelled on the Recommended Food Score and a lower ARFS has previously shown an association with higher self-reported health service usage [13]. This highlights how the ability of the tool to capture diet comprehensively and accurately as well as the age of the cohort, the study outcomes chosen and having objective data throughout the length of the follow-up period over which the outcome is measured, are important. 

## 11. Conclusions

Mid-aged women with lower dietary variety and quality, as measured by the ARFS, do not have increased cumulative Medicare costs, but do have a greater number of Medicare claims, albeit of a lower cost per claim. The relationship between diet quality and cumulative health care costs and claims is not consistent in the medium term. This suggests a need to monitor the Charge, Benefit and Gap as well as the number of claims over time to further examine health care costs in association with diet quality. In particular, future research could address whether there is eventually a monetary saving to Medicare or to the women personally, as measured by the Gap payment, in the long-term for those with dietary patterns that adhere more closely to national recommendations. 
